# Synthesis and Adsorption Properties of Novel Bacterial Cellulose/Graphene Oxide/Attapulgite Materials for Cu and Pb Ions in Aqueous Solutions

**DOI:** 10.3390/ma13173703

**Published:** 2020-08-21

**Authors:** Shen Song, Zhao Liu, Ji Zhang, Caizhen Jiao, Ling Ding, Shengrong Yang

**Affiliations:** 1 College of Chemistry & Chemical Engineering, Northwest Normal University, Lanzhou 730070, China; 2016118076@nwnu.edu.cn; 2 New Rural Development Research Institute of Northwest Normal University, Northwest Normal University, Lanzhou 730070, China; xncfzyjynwnu@163.com (C.J.); 2017222068@nwnu.edu.cn (L.D.); 3 Department of Orthopaedic Surgery, Orthopaedic Institute, The First Affiliated Hospital, Soochow University, Suzhou 215006, China; zhaoliu@suda.edu.cn; 4 Lanzhou Institute of Chemical Physics, Chinese Academy of Sciences, Lanzhou 730000, China

**Keywords:** bacterial cellulose, graphite oxide, attapulgite, adsorption, heavy metal ions

## Abstract

Removing heavy metal ions from industrial wastewater is one of the most important and difficult areas of the water treatment industry. In this study, Bacterial Cellulose/Polyvinyl Alcohol/Graphene Oxide/Attapulgite (BC/PVA/GO/APT) composites were successfully prepared via a repeated freeze-thaw method using bacterial cellulose, polyvinyl alcohol as the skeleton, and graphene oxide, attapulgite as fillers. The capacities of adsorbing Cu^2+^ and Pb^2+^ ions in solution were investigated. FTIR, XRD, SEM, BET, and TG-DSC analyses showed that the BC/PVA/GO/APT hydrogel has a better hydrophilicity, a larger specific surface area and a better thermal stability than traditional materials. We found that the adsorption of Cu^2+^ and Pb^2+^ ions can be accurately predicted by the Freundlich kinetic model, and the optimal adsorption capacities of these ions were found to be 150.79 mg/g and 217.8 mg/g respectively. Thermodynamic results showed that the adsorption process is spontaneous and exothermic. BC/PVA/GO/APT composites are suggested to be an ideal adsorption material for removing heavy metal ions from industrial wastewater.

## 1. Introduction

With the development of human society, the discharging of industrial wastewater has increasingly caused serious problems that are harmful to both human health and the water ecosystem [1,2]. Excessive enrichment of metals such as Cu (II) and Pb (II) in human body can lead to serious damage to different organs and diseases of cardiovascular, nervous system and immune system [3,4]. Typical methods such as adsorption, filtration, biological treatment, chemical degradation, and membrane separation are used to treat industrial sewage [5,6,7]. Moreover, adsorption has been identified as one of the most cost-effective treatments due to its unique advantages such as simple operation, low cost and high removal rate for metal ions [8,9,10].

Commonly used adsorption materials include activated carbon, natural minerals, resins, natural polymers, industrial and agricultural wastes, and metal oxide nanomaterials etc. In recent years, the demands for biomass adsorbents [11] such as chitosan [12,13], cellulose [14,15] and various lignocellulosic wastes [16] for renewable, biodegradable and biocompatible materials have increased. One of the most important biomass adsorbents is bacterial cellulose (BC), a biopolymer with the same molecular structure unit as natural plant cellulose [17]. Besides the properties for industrial applications such as industry high purity, unique three-dimensional (3D) cross-linking network and high specific surface area, BC is also porous, degradable and does not contribute to secondary pollution [18,19,20]. Therefore, a large number of BC composites have been widely used in a variety of areas, such as for optical transparent films, photocatalytic nanomaterials, and biomedical and adsorption materials (especially dyes and heavy metals) [21,22]. Using cellulose as an adsorbent directly has a small adsorption capacity and low selectivity. In order to improve the adsorption capacity of cellulose, it is necessary to modify the cellulose structure or prepare composite materials. One of the most notable BC composites is polyvinyl alcohol (PVA), which has strong hydrophilic properties and good biological compatibility and can be used to form a high strength hydrogel by the freeze-thaw cycle without adding a crosslinking agent [23]. This may be due to the large number of hydroxyl groups present in the main chain of PVA that can be crosslinked to form hydrogen bonds, a phenomenon conducive to the synthesis of hydrogel complexes [24]. However, a study on the preparation of composites by cross-linking of bacterial cellulose and polyvinyl alcohol for adsorption of heavy metal ions has not been reported before.

GO (graphene oxide) is a highly oxidized derivative of graphene, which has attracted great attention since it was first discovered in 2004 [25]. In recent years, one of the important applications of graphene oxide composites is to remove heavy metal ions from wastewater by adsorption [26]. Results showed that the adsorption properties of these composites for heavy metal ions can be significantly improved by adding a small amount of graphene oxide [27,28,29]. Attapulgite (APT) is a special silicate clay mineral consisting of two continuous tetrahedral sheets and a discontinuous octahedral sheet group [30]. This special crystal morphology with nanopores enables this low cost, stable and environmental friendly clay mineral to capture various pollutants such as dyes, heavy metal ions, etc. [31,32]. functionalized halloysite nanotubes enhanced removal of lead (II) ions from aqueous solutions [3]. Some APT composites polymer materials were produced to adsorb heavy metal ions (Cu^2+^ and Cd^2+^) and methylene blue [33,34]. The combination of GO and ATP as filler in hydrogel for heavy metal ions or dye adsorption has not been reported. Therefore, in NaOH urea green solvent system, the composite aerogels were prepared by adding GO and ATP into the three-dimensional network structure of bacterial cellulose polyvinyl alcohol by means of the dissolution freeze thaw cycle method. This simple and efficient method for the removal of heavy metal ions in wastewater can be applied for the preparation of environmentally friendly composite materials.

## 2. Materials and Methods

### 2.1. Materials

BC was synthesized by *Gluconacetobacter xylinum* (ATCC53582, Wuhan, China) in Hestrin and Schramm (HS) static cultures [35], GO was obtained by using the Hummers method [36], and APT was prepared at the Lanzhou Institute of Chemical Physics [37], Chinese Academy of Sciences. PVA, CuSO_4_·5H_2_O and Pb(NO_3_)_2_ were purchased from the China National Medicine Reagent Co., Ltd. (Shanghai, China). Other chemical reagents and pharmaceuticals used in this study were of analytical grade. All solutions were prepared with distilled water.

### 2.2. Preparation of Composite Materials

Firstly, 2.0 g of BC powder was gradually dissolved in a 100 mL NaOH/urea/H_2_O (7:12:81) [38] solution at −12 °C by vigorous magnetic stirring. After that, 1.0 g of PVA was added into the mixture, stirred and dissolved uniformly, followed by 50 mg of GO and various amounts of APT (1.0 g, 1.5 g and 2.0 g); bubbles were removed by ultrasonic treatment. The uniformly mixed polymer was poured into a 12-well culture plate for three freeze-thaw cycles (12 h at −20 °C and 4 h at room temperature) to form a columnar composite adsorbent. In order to remove NaOH and urea, the composite materials were washed in distilled water until the pH returned to 7.0. The processed samples were labeled as BP(BC/PVA), BPGA1%, BPGA1.5%, and BPAG 2% (corresponding to the different APT concentrations), and subsequently dried in a vacuum-freeze dryer at −65 °C for 72 h before further analysis.

### 2.3. Characterization

A Fourier-transform infrared spectrometer recorded FTIR spectra of the samples in the range of 4000 cm^−1^–400 cm^−1^ (Is10, Thermo Corp., Waltham, MA, USA) by the standard KBr pressed-pellet method, and 16 scans were performed at 4 cm^−1^ resolution. The crystallinity degree of the samples was determined via X-ray diffraction (D8 Advance, Bruker, Germany) at an accelerating voltage of 40 kV and current of 150 mA. The scanning speed was set as 5°/min and the diffraction angle (2*θ*) was varied between 5° and 50°. The surface morphology and chemical composition of the samples were characterized with scanning electron microscopy (SEM) and energy dispersive spectrometry (EDS) (Zeiss ULTRA Plus, Heidenheim, Germany). Nitrogen adsorption/desorption isotherms were collected on a Tristar 3020 apparatus (Micromeritics, Norcross, GA, USA) at −196 °C. The specific surface areas were calculated using the Brunauer-Emmett-Teller (BET) method, and the pore size distributions (PSD) were calculated from the desorption branch of the isotherm with the Barrett-Joyner-Halenda (BJH) mode [39,40,41,42]. The thermal stability of the samples was characterized by using a synchronous thermal analyzer (STA449C, NETZSCH, Selbu, Germany) with a 10 °C/min heating rate from room temperature to 500 °C in an N_2_ atmosphere.

### 2.4. Cu^2+^ and Pb^2+^ Adsorption by the Composite Hydrogels

#### 2.4.1. Adsorption Kinetics

Adsorption process of Cu and Pb ions was carried out separately. Dried samples (10 mg) were immersed into a 10 mL Cu^2+^ and Pb^2+^ solution (200 mg/L) at room temperature. The concentration of the solution was measured at a certain time interval with an atomic absorption spectrophotometer (WFX-210, Shanghai Husi Experimental Instrument Co., Ltd., Shanghai, China). The adsorption capacity of samples at time t ($Q_{t}$, mg/g) and the equilibrium adsorption capacity ($Q_{e}$, mg/g) were calculated according to following equations (Equations (1) and (2)):

(1) $$Q_{t}=\left(C_{0}-C_{t}\right)V/M$$

(2) $$Q_{e}=\left(C_{0}-C_{e}\right)V/M$$

where $C_{0}$ indicates the initial concentration, $C_{t}$ represents the concentration of the solution at time t, $C_{e}$ (mg/L) represents the equilibrium concentration, and V (L) and M (g) indicate the volume of solution and the mass of dry sample, respectively.

#### 2.4.2. Effect of pH and Initial Concentration

The responses of the samples on varying initial concentration and pH of Cu^2+^ and Pb^2+^ solutions have been investigated using the method above. The pH values (adjusted by using diluted HCl or NaOH solutions) were measured to be 2, 3, 4, 5, and 6, while the concentrations of Cu^2+^ and Pb^2+^ solutions were changed from 50 to 250 mg/L.

#### 2.4.3. Reusability of the Hydrogels

The adsorbents with copper and lead ions were stirred with 20 mL 0.2 mol/L EDTA-Na solution for 3 h. After that, the stirred copper and lead ions were washed twice with distilled water and subsequently were used in the adsorption/desorption cycle experiment. Four tests were carried out according to the method described in Section 2.4.1.

## 3. Results and Discussion

### 3.1. FTIR Analysis

**Figure 1:**
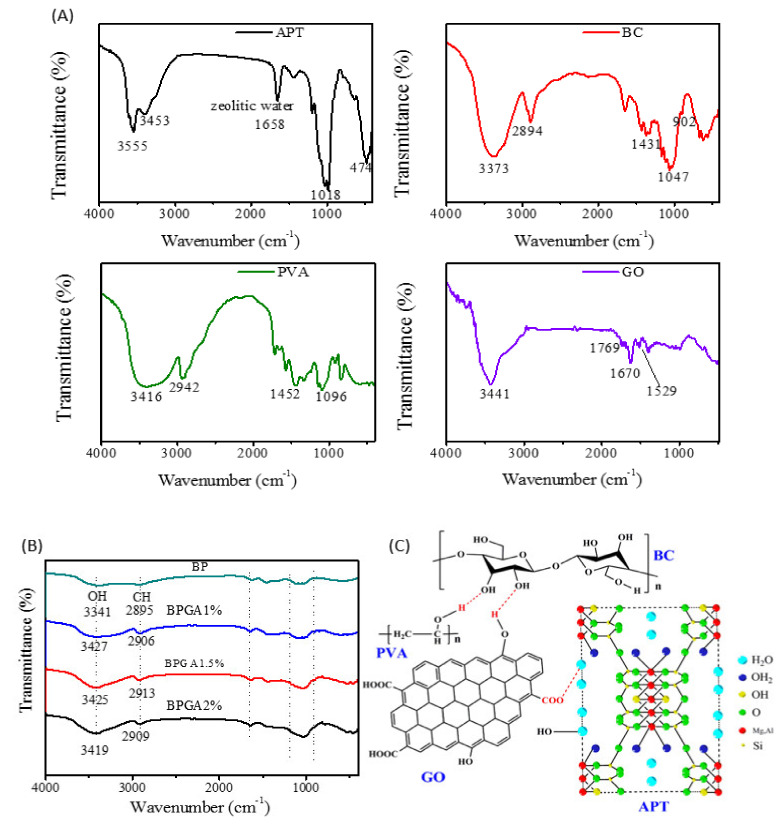
The FTIR spectra of initial components and prepared adsorbent materials. (**A**) represents the FTIR spectra of initial components (APT, BC, PVA, and GO) and (**B**) represents the prepared adsorbent materials (BP, BPGA1%, BPGA1.5% and BPGA2%), (**C**) represents a schematic diagram of the hydrogen bonding of the initial components.

The initial components and composite components were characterized using FTIR. Obtained spectra are plotted in Figure 1.

According to Figure 1A, the infrared spectrum of APT shows that the peaks at 3555 cm^−1^ and 3453 cm^−1^, which correspond to the stretching vibrations of Al–OH and Mg–OH, respectively, while the peaks at 1018 cm^−1^ and 474 cm^−1^ correspond to the Si–O and Si–O–Si bonds [43].

For BC, the broad peak at 3373 cm^−1^ is attributed to the stretching vibrations of intermolecular and intramolecular groups. The peaks at 2894, 1047 and 1431 cm^−1^ correspond to the bending vibration modes of CH_2_–CH, C–O and C–H respectively, while at 902 cm^−1^ the characteristic peak of the glycosidic bond is seen [44]. The FTIR spectrum of PVA shows a broad peak at 3416 cm^−1^, which is attributed to the O–H stretching vibration. The peaks at 2942, 1452 and 1096 cm^−1^ correspond to the expansion and contraction of C–H and C–O–C bonds and to the bending vibration mode of C–H respectively [45]. In the last frame of Figure 1A, the FTIR spectrum of GO shows the characteristic peaks of OH (3420–3606 cm^−1^), –C=O– (1750–1850 cm^−1^), –COOH (1650–1750 cm^−1^), and C=C (1500–1600 cm^−1^) [46]. As it can be seen in Figure 1B, the characteristic peak of BP at 3341 cm^−1^ corresponds to the stretching vibration of the intermolecular and intramolecular hydrogen bonds between OH groups in BC and PVA. Comparing the spectra of the initial components and BP shows that no new characteristic peaks are found for the composite after introducing GO and APT. However, the characteristic peak of CH_2_–CH (2895 cm^−1^) in BC overlaps with the C–H peak in PVA. After adding GO and APT, the OH peak broadens and shifts to slightly lower wavenumbers. The results indicate that hydrogen bonds are formed between polymers as shown in Figure 1C and contribute to the interactions of polymers.

### 3.2. XRD Analysis

**Figure 2:**
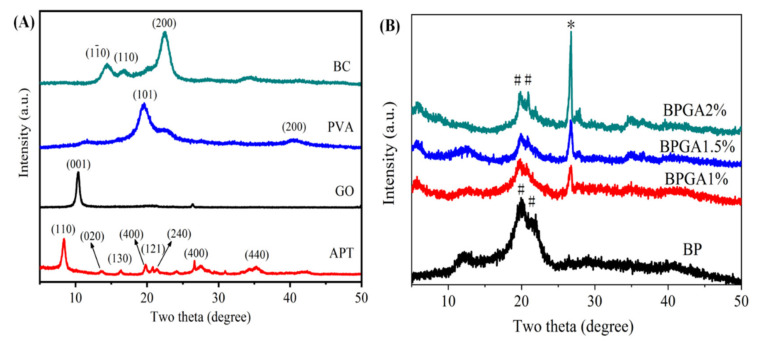
The X-ray diffraction spectra of initial components and prepared adsorbent materials. Frame (**A**) indicates the X-ray diffraction spectra of initial components (BC, PVA, APT and GO) and (**B**) indicates the X-ray diffraction spectra of prepared adsorbent materials (BP, BPGA1%, BPGA1.5% and BPGA2%), (*) APT, (#) BC.

Figure 2 shows the XRD patterns of the initial components (BC, PVA, APT, and GO) and composites (BP, BPGA1%, BPGA1.5%, and BPGA2%).

One can distinguish in the XRD spectrum of BC that there are noticeable peaks at 2θ = 14.30°, 16.70° and 22.66°, indicating that BC has a typical crystalline form of cellulose I [47], which is attributed to the typical reflection planes (1$\bar{1}$0), (110), and (200) of cellulose I structure (JCPDS NO. 50-2241). The XRD pattern of PVA shows a strong diffraction peak at 2θ = 19.7° and 40.6° [48,49]), indexing to the (101) and (200), which corresponds to the crystalline PVA (JCPDS: 53-1487). The XRD spectrum of APT shows peaks at 2θ = 8.4°, 13.64°, 16.46°, 19.78°, 20.86°, 21.39°, 27.48° and 35.34°. These are the characteristic crystallization peaks corresponding to crystal faces: (110), (200), (130), (040), (121), (240), (400), and (311) [50], which match well with the standard attapulgite patterns of JCPDS card No. 21-0958. GO exhibits a strong diffraction peak at 2θ = 10.3° due to the π-π interaction and irregular packing of hydrogen bonds, which can be indexed as the (001) reflection of the GO (PDF-ICDD 41-1487). As shown in Figure 2B, the characteristic peaks of GO disappear upon the formation of the BPGA1%, BPGA1.5% and BPGA2% composite materials, indicating that GO exists in a single layer structure in the composite, which is in accordance with results previously reported in literature [51,52].

Moreover, the three-dimensional network structure of BC promotes the expansion and extension of the GO layer, reducing wrinkles and the multi-layer aggregation of GO. According to observed XRD spectra of the composites, the peak at 2θ = 27.48° in the spectra of BPGA1%, BPGA1.5% and BPGA2% becomes stronger with the increase of APT content, corresponding to the characteristic diffraction peak of APT. Compared with BP, the intensity of the main diffraction peak in the composites at 2θ = 19.95° is significantly reduced and the characteristic peak of cellulose is significantly weakened and shifted to 2θ = 21.25°. This suggests that the crystallinity of BC in the composite formed by the combination of GO and APT decreases after dissolution regeneration. Combined with FTIR analysis, the existence of hydrogen bonding and interactions between BC, PVA, GO, and APT has been inferred [53].

### 3.3. TG-DSC Analysis

**Figure 3:**
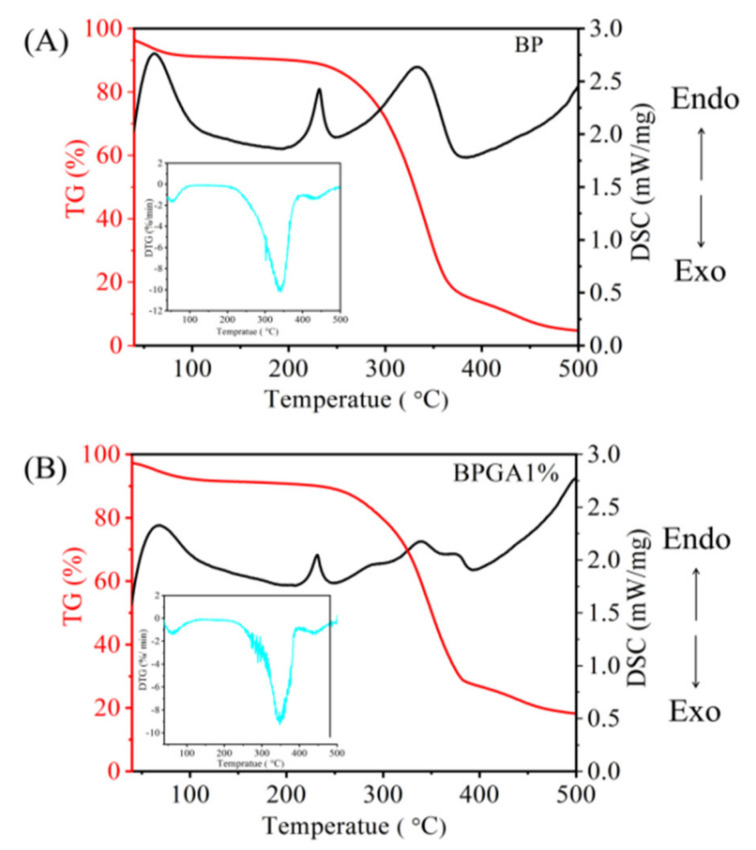
Frames (**A**,**B**) show the thermal characteristics of the prepared adsorbent materials, BP and BPAG 1%, based on TG-DSC and DTG (insets).

Figure 3A,B indicates the TG-DSC curves of the backbone material (BP) and the BPGA1% composite material after introducing GO and APT respectively.

The TG curves indicate the thermal decomposition of BP and BPGA1% can be divided into three phases. The weight loss for temperatures below 100 °C mainly due to the evaporation of free water, physically adsorbed water and bound water in the sample. The pyrolysis section between 250 and 375 °C is the main phase of weight loss. The carbonization of BC and PVA into CO and CO_2_ results in 83.19% weight loss, including destroying the long-chain cellulose structure, the main chain of cellulose and the dehydration cleavage of the upper glucose unit. In addition, when the temperature is close to 375 °C, breaking of the glycoside bonds, C–O bonds and C–C bonds occurs. The remaining structure forms a carbon residue during the pyrolysis stage between 375 and 500 °C and the final mass is maintained at 4.72%. Compared with the case of BP, the TG curve of the BPGA1% composite, after the introduction of GO and APT, indicates differences in the mass-loss rate. The mass loss is around 7.92% at the first phase (below 100 °C) due to evaporation of free moisture, while the mass loss reaches 70.66% during the second phase of mass loss (250 to 375 °C). In contrast to BP, the introduction of GO and APT increases the thermal stability of composite and reduces its rate of pyrolysis. During the third phase (375 to 500 °C), the pyrolysis rate of the composite material is further slowed down.

The DSC curves of BP and BPGA1% were observed as well. Below 100 °C, there was a strong adsorption heat peak of adsorbed water, which was corresponding to the mass loss in the first stage of TG curve. There are two strong endothermic melting peaks in the DSC curve of BP between 200 °C and 400 °C, which is consistent with the main mass loss of the skeleton material BP in the second stage. There is a weak endothermic peak between 200 °C and 400 °C in BPGA1%, and there is no obvious glass transition temperature. This shows that the introduction of GO and APT enhances the physical binding point of the mixed system, and that the thermodynamic properties change upon adding GO and APT. The main peak of DTG curve corresponds to the pyrolysis temperature of endothermic peak of DSC curve.

### 3.4. SEM-EDS Characterization

**Figure 4:**
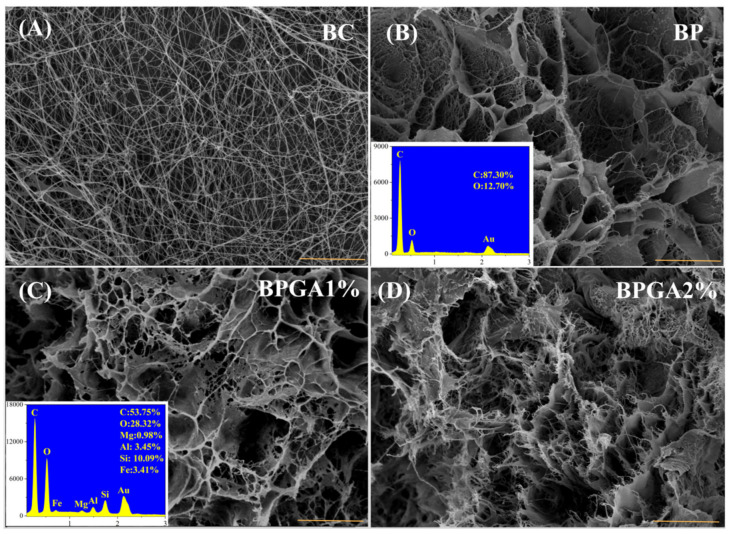
SEM images of the prepared adsorbent materials, (the insets are the EDS spectra with a scale bar of 5 μm). (**A**) BC, (**B**) BP, (**C**) BPGA1%, (**D**) BPGA2%.

The microscopic morphology of the sample was characterized by SEM, the atomic species and elemental percentages in the samples were analyzed by EDS. Figure 4A illustrates the microscopic morphology and fiber network structure of pure BC, where uneven pores can be clearly observed. Since it statically ferments, the fiber tends to be chaotic.

Figure 4B illustrates the microscopic morphology of BC bound to PVA after dissolution regeneration: After dissolution and regeneration, the fiber network structure of BC is interweaved with PVA to form uneven honeycomb-like pores. The inset displays the elements in the BP sample and their corresponding percentages as measured with EDS spectrum. Figure 4C,D shows SEM images for the cases of added GO and different amounts of APT. It can be noted that two-dimensional GO and one-dimensional APT are interwoven with three-dimensional BC fibers to form a dense, uneven mesh. Moreover, cracks are also evident because of the structure of fibers and the rougher surface of composite. The zoom in SEM images of the BPGA1% and BPGA2% composites reveals a slight difference. The main reason is because the pore structure of the composite contains more zigzags and wedges with an increased APT content, and the surface structure becomes more irregular.

### 3.5. BET Analysis

**Figure 5:**
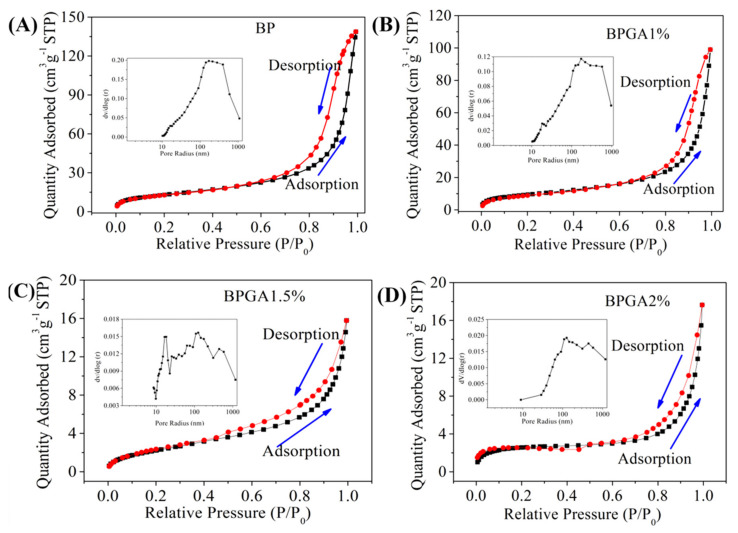
The N_2_ adsorption/desorption isotherms of the prepared adsorbent materials: BP, BPGA1%, BPGA1.5% and BPGA2%; the insets indicate the corresponding pore size distributions. (**A**) BP, (**B**) BPGA1%, (**C**) BPGA1.5%, (**D**) BPGA2%.

The specific surface area has an important influence on the performance of an adsorption materials. In general, the specific surface area of adsorption material is positively related to the adsorption capacity, although the latter may be affected by a number of factors such as the surface properties of the adsorbent itself, i.e., the type of functional groups and whether the material matches the adsorbed molecules. The higher the specific surface area and porosity, the stronger the adsorption capacity. The special active chemical functional groups such as amino group, amide bond and hydroxyl group distributed on the surface of the adsorbent can form stable chelates with heavy metal ions and enhance the adsorption and chelation capacity. Therefore, the adsorption capacity increases with an increase of specific surface area. Figure 5 shows the N_2_ adsorption-desorption curves of the initial material (BP) and BPGA1%, BPGA1.5% and BPGA2% composites along with the pore diameter distribution curve.

As it can be seen in Figure 5, according to the IUPAC classification [54], an H3-type hysteresis loop in the type IIb isotherm is observed in all samples. This type of isotherm is the most described phenomenon in the BET formula, and represents the physical adsorption process on non-porous or macroporous adsorbents. The H3 hysteresis loop corresponds to the SEM image and the surface of the composite shows an irregular and flat slit structure, crack or wedge structure. The calculated specific surface areas of BP, BPGA1%, BPGA1.5%, and BPGA2% composites with the BET formula are 47.35 m^2^g^−1^, 34.08 m^2^g^−1^, 8.78 m^2^g^−1^, and 8.47 m^2^g^−1^ respectively. The calculated pore volumes with BJH model are 0.215 cm^3^g^−1^, 0.153 cm^3^g^−1^, 0.024 cm^3^g^−1^, and 0.027 cm^3^g^−1^ respectively. Compared with BP, the specific surface area of BPGA 1% decreased after the introduction of GO and APT, and the specific surface area of BPGA 1.5% decreased further due to the increase of APT content, which indicates that two-dimensional GO and one-dimensional APT are fully dispersed in the 3D structure of BC, and the inner big large pores are filled to form small holes, resulting in a decrease of pore size and volume, a decrease of specific surface area, and an increase of active sites and driving force [55]. In the high relative pressure range of 0.8–1, BP and BPGA1% hysteresis ring, indicating that a large number of mesopores are evenly distributed in the material [56], which is consistent with the description result of the pore distribution figure in Figure 5.

### 3.6. The Adsorption of Cu^2+^ and Pb^2+^ Ions by the Composite Materials

#### 3.6.1. The Effect of pH on the Adsorption Performance

**Figure 6:**
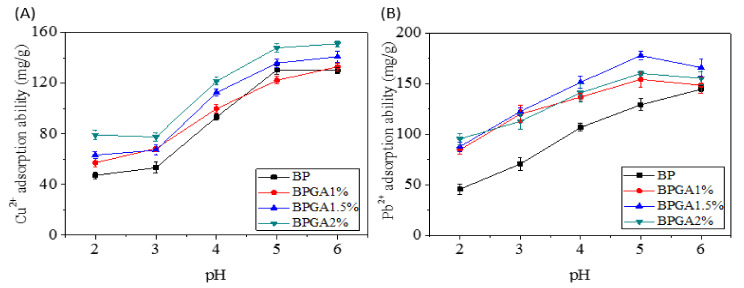
The effect of pH on the adsorption performance for Cu^2+^ ions (**A**) and Pb^2+^ ions (**B**).

The pH has an important influence on the adsorption and reaction rate of the adsorbent surface. Figure 6A,B shows that the adsorption capacity increases as the increased pH of the solution increases.

The best adsorption capacity is observed at pH = 6 for Cu^2+^ and pH = 5 for Pb^2+^ respectively. The H^+^ ions compete with metal ions (Cu^2+^ and Pb^2+^) for adsorption sites at low pH. The combination between H^+^ ions and metal ions is decreasing with increased pH while the combination between O^−^ ions and metal ions is enhancing. Therefore, the positive metal ions are adsorbed by the negative adsorbent (BP) in the solution. Due to the addition of GO and APT, more adsorption functional groups are introduced into the composite adsorbent. At pH < 2.5, GO and APT form heteropolymers and the charge properties of the surface of APT are changed by GO; thus, the surfaces of BPGA1%, BPGA1.5% and BPGA2% composite adsorbents are negatively charged. In addition, H^+^ ions are bonded to Si (Al)–O– groups at low pH, and their bonding to metal ions is reduced. Therefore, the capacity for metal ions in the composite is reduced at pH < 3. As the pH increases from 2.5 to 6.0, the carboxyl groups on the GO gradually deprotonate, resulting in a more hydrophilic surface and less APT aggregation. GO has a positive effect on APT and the negative charge on the surface of composite adsorbents (BPGA1%, BPGA1.5% and BPGA2%) becomes stronger, resulting in a strong electrostatic attraction between metal ions (Cu^2+^ and Pb^2+^) and adsorbent. In addition, with the increase of pH, the binding of Si (Al)–O– group to H^+^ is weakened, while the binding of metal ions (Cu^2+^ and Pb^2+^) is enhanced. Therefore, more metal ions are adsorbed onto the composite adsorbent at higher pH [57].

#### 3.6.2. Adsorption Kinetics

Adsorption kinetics is an important means to understand the potential adsorption mechanisms and determine the potential control-rate steps. In addition, the adsorption mechanism is determined by the chemical and physical properties of adsorbent and mass transfer rate of adsorbate onto the adsorbent surface. Therefore, the following kinetic model and calculation method are applied to establish the adsorption mechanism, (Equations (3) and (4)):

(3) $$\log\left(Q_{e}-Q_{t}\right)=\log Q_{e}-k_{1}t/2.303$$

(4) $$t/Q_{t}=1/k_{2}Q_{e}^{2}+t/Q_{e}$$

where $Q_{e}$ (mg/g) and $Q_{t}$ (mg/g) are the adsorption capacities of hydrogels at the adsorption equilibrium and time t. $k_{1}$ (min^−1^) and $k_{2}$ (mg/g min^−1^) are the rate constants of the pseudo-first order and pseudo-second-order kinetic models, respectively.

**Figure 7:**
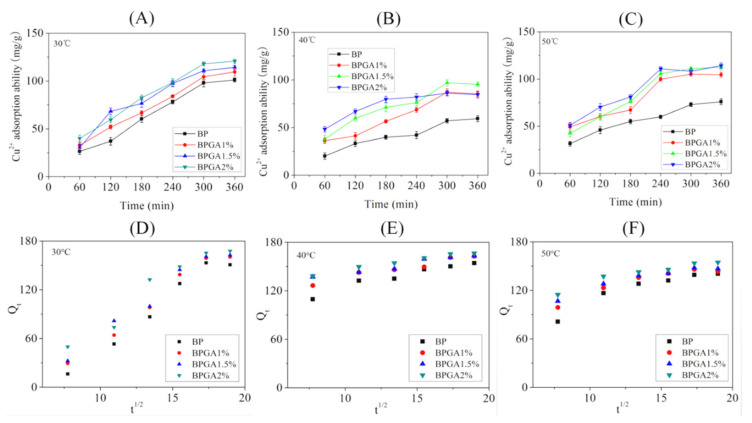
The adsorption kinetics of Cu^2+^ onto the prepared adsorbent materials at 30 °C (**A**), 40 ℃ (**B**) and 50 °C, (**C**) intraparticle diffusion models for the adsorption of Cu^2+^ at 30 °C (**D**), 40 °C (**E**), and 50 °C (**F**).

**Figure 8:**
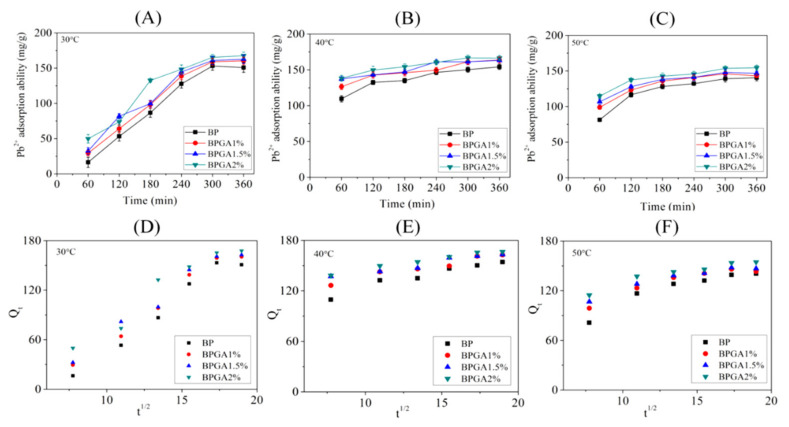
The adsorption kinetics of Pb^2+^ onto the prepared adsorbent materials at 30 °C (**A**), 40 °C (**B**) and 50°C (**C**); intraparticle diffusion models for the adsorption of Pb^2+^ at 30 °C (**D**), 40 °C (**E**) and 50 °C (**F**).

Batch experiments were carried out at different temperatures (30 °C, 40 °C and 50 °C) according to the method described in Section 2.4.1. Figure 7 and Figure 8 show the graphs and kinetic fits of the adsorption capacities of Cu^2+^ and Pb^2+^ ions over time at different temperatures.

Figure 7A–C presents the change in the adsorption capacity for Cu^2+^ ions over time at different temperatures. In general, the adsorption capacity increases rapidly in the early stage, slows down as time progresses, and reaches equilibrium at last. A reasonable explanation may be that the material provides a large number of adsorption sites in the initial stage, which leads to a rapid rise of adsorption rate. When the available adsorption sites are gradually occupied by Cu^2+^ ions, the adsorption rate slows down and reaches equilibrium. In addition, the adsorption capacity decreases as temperature increases from 30 °C to 40 °C and subsequently shows a slight increase as temperature increases from 40 °C to 50 °C. The maximum adsorption capacity is found at 30 °C. The adsorption capacity of BPGA2% at 30 °C, 40 °C and 50 °C was 120.79 mg/g, 86.06 mg/g and 114.15 mg/g respectively. This indicates that the temperature has an inhibitory effect on the performance of the adsorbent during the adsorption process.

Table 1 shows the fitting values of three kinetic model parameters; R^2^ values indicate that the particle diffusion model fit (R^2^ > 0.99) is better than the quasi-first-order kinetics fit (R^2^ > 0.87) and the quasi-second-order kinetic model fit, which means the intraparticle diffusion model most accurately describes the adsorption mechanism of Cu^2+^ ions.

The particle diffusion model of Cu^2+^ is multi-linear, indicating that adsorption is achieved via several steps. Firstly, the adsorbate diffuses from the solution to the external surface of the adsorbent, causing a few active sites to be occupied by Cu^2+^. Subsequently, Cu^2+^ is gradually adsorbed during the intraparticle diffusion phase. Finally, the number of adsorption sites decreases and the intraparticle diffusion rate begins to slow down during the adsorption equilibrium phase. In addition, the $K_{T}$ value at 30 °C suggests that the fastest migration rate of Cu^2+^ ions is from the solution to adsorbent surface. Moreover, the model curve of particle diffusion does not pass through the origin, indicating that the total adsorption kinetics of the adsorbent material are mainly controlled by surface diffusion and intraparticle diffusion [58].

Figure 8 shows the adsorption capacity of the adsorbents for Pb^2+^ over time. Clearly, the adsorption capacity for Pb^2+^ at different temperatures (30 °C, 40 °C and 50 °C) increases rapidly in the initial stage of adsorption, then the adsorption rate slows down to equilibrium. These results indicate that there is a large attraction between adsorbent and Pb^2+^ ions in the initial adsorption stage. Temperature accelerates the diffusion rate of Pb^2+^, resulting in changes of adsorption capacity. Combined with calculated kinetic data in Table 2, the R^2^ values of the particle diffusion model and the quasi-first-order kinetic model fits are relatively high, while the particle diffusion model fits the best (R^2^ > 0.99).

According to the plots of $Q_{t}$ versus $t^{1/2}$ in Figure 8, frames (D)–(F). In fact, there are two stages with obvious inflection points: the initial phase is the adsorption on the outer surface of the adsorbent caused by the diffusion effect of the boundary layer; and the second phase tends to be gentle, which means that the adsorption is mainly in the diffusion stage before reaching equilibrium [59]. In addition, the specific internal diffusion and external mass transfer occur simultaneously, cooperating with the adsorption process.

#### 3.6.3. Effect of the Initial Concentration of the Cu^2+^ and Pb^2+^ Solution on the Adsorption Performance

**Figure 9:**
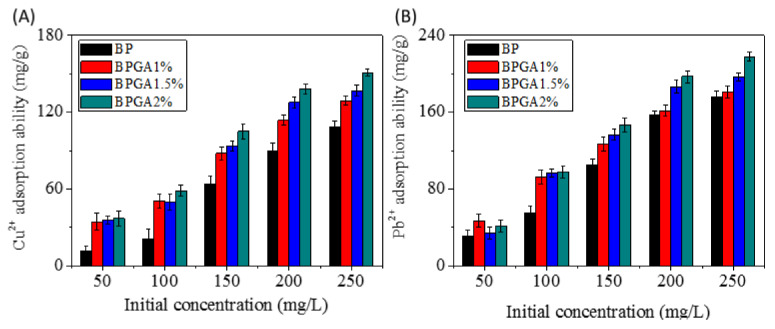
Effect of the initial metal ion concentration on the adsorption performance. (**A**) Cu^2+^, (**B**) Pb^2+^.

The initial concentration of heavy metal ions has significant influence on the adsorption capacity. A test has been carried out with 10 mL Cu^2+^ and Pb^2+^ ion solutions of different concentrations (50–250 mg/L) with 10 mg adsorbent. As shown in Figure 9, it is apparent that the adsorption capacity is affected by the initial concentration of Cu^2+^ and Pb^2+^ solutions.

The adsorption capacity increases for higher initial concentrations, but this increase stops above a concentration of metal ions of 250 mg/L, which means that few adsorption sites remain on the surface and the adsorption almost reaches equilibrium. This phenomenon may be because the initial concentration provides a driving force to overcome the mass transfer limitation of Cu^2+^ and Pb^2+^ between the liquid and solid phases. The higher the initial concentration of the Cu^2+^ and Pb^2+^ solution, the stronger the driving force and the higher adsorption capacity that will be found. Compared with BP, BPGA 1% and BPGA 1.5%, BPGA 2% has the best adsorption performance. The adsorption capacity of BPGA2% composite in Cu^2+^ and Pb^2+^ solutions (250 mg/L concentration) was 150.79 mg/g and 217.81 mg/g, respectively. Compared with BC, GO and ATP composites, the adsorption capacity was significantly improved. The maximum adsorption capacities of some materials for heavy metal ions are shown in Table 3.

#### 3.6.4. Study of the Moderate-Temperature Adsorption Model of the Cu^2+^ and Pb^2+^ Ion Adsorption Process

In order to understand the adsorption behavior of Cu^2+^ and Pb^2+^ ions on the sample adsorbent materials, the following isothermal adsorption model was applied and the calculated results (Equations (5)–(7)) are shown in Table 4.

(5) $$\ln Q_{e}=\ln K_{F}+1/n\ln C_{e}$$

(6) $$Q_{e}=RT/b_{T}\left(\ln K_{T}+\ln C_{e}\right)$$

(7) $$C_{e}/Q_{e}=C_{e}/Q_{max}+1/K_{L}Q_{max}$$

where $C_{e}$ (mg/L) and $Q_{e}$ (mg/g) represent the equilibrium heavy metal ions concentration and equilibrium adsorption capacity respectively. $K_{F}$ ((mg/g) (L/mg)^1/n^) and 1/n are the Frenchmen constants, which are related to the binding energy and adsorption strength. R is the gas constant (8.314 J/(mol∙K)), $K_{T}$ (L/g) is the equilibrium binding constant and $Q_{max}$ (mg/g) is the maximum monolayer covering capacity. $b_{T}$ (J/mol) is associated with adsorption heat and $K_{L}$ (L/mg) is the Langmuir constant associated with the affinity of binding sites, obtained by computation. An important characteristic of the Langmuir isotherm model is described by a dimensionless constant, the Hall separation factor ($R_{L}$), and is shown in the following form, (Equation (8)):

(8) $$R_{L}=1/\left(1+K_{L}C_{0}\right)$$

where $C_{0}$ (mg/g) is the initial concentration of the solution, and $R_{L}$ is an index to indicate whether the adsorption isotherm is favorable. Adsorption is favorable if $0<R_{L}<1$, and the adsorption is linear if $R_{L}=1$. If $R_{L}>1$, adsorption is unfavorable, and if $R_{L}=0$, the adsorption process is irreversible [64].

It can be observed from Table 4 that the adsorption data for Cu^2+^ and Pb^2+^ ions are relatively low in the Langmuir isothermal model. We note that the dimensionless separation factor $R_{L}$, which describes the Langmuir isotherm characteristics, predicts the affinity between adsorbate and adsorbent. The $R_{L}$ values decrease with the metal ion concentration, which suggests that the affinity between adsorbent and adsorbate is able to become stronger at higher concentrations of metal ions. Taking adsorption at 200 mg/L of initial concentration: For Cu^2+^, the values of RL for BPGA1% and BPGA1.5% were 0.4274 and 0.4348. The values of RL for BPGA1%, BPGA1.5% and BPGA2% were 00506, 0.1329 and 0.0512 for Pb^2+^. This conclusion is consistent with the dependence of the adsorption capacity of Cu^2+^ and Pb^2+^ ions on the initial solution concentration. At the same time, it also explains why the adsorption capacity of the prepared adsorbent material is higher for Pb^2+^ ions than that for Cu^2+^ ions. The best R^2^ value (R^2^ > 0.98) for Cu^2+^ and Pb^2+^ ions is for the Freundlich isothermal model, which is based on heterogeneous adsorption and indicates that the adsorbent and heavy metal ions are multi-layered. Furthermore, the fitted n value (>1 or ≤1) indicates that the adsorption process is accompanied by continuous or intermittent adsorption. The Temkin model is commonly used to describe heterogeneous surface energy systems. According to the $b_{T}$ values of Cu^2+^ and Pb^2+^ ions shown in Table 4, it can be concluded that the physical adsorption process of the prepared adsorbent for metal ions is more effective than chemisorption process [57].

#### 3.6.5. Analysis of the Thermodynamic Parameters during Adsorption of Cu^2+^ and Pb^2+^ Ions

In order to investigate the effect of temperature on adsorption, changes in the Gibbs free energy ($\Delta G^{0}$), enthalpy ($\Delta H^{0}$) and entropy ($\Delta S^{0}$) during the process are calculated according to the following equations, (Equations (9)–(11)):

(9) $$K_{d}=Q_{e}/C_{e}$$

(10) $$\Delta G^{0}=-RT\log K_{d}$$

(11) $$\log K_{d}=\Delta H^{0}/R_{T}+\Delta S^{0}/R_{T}$$

where $C_{e}$ (mg/L) and $Q_{e}$ (mg/g) are the equilibrium heavy metal ions concentration and equilibrium adsorption capacity. R and T (K) are the gas constant (8.314 J/(mol∙K)) and the absolute temperature expressed in kelvin. The calculated thermodynamic quantities are shown in Table 5.

The negative value of the Gibbs energy change (ΔG^0^) at three different temperatures reveals the spontaneity and thermodynamics of Cu^2+^ and Pb^2+^ ions adsorbed onto the prepared adsorbent material. As temperature increases, an increase in the Gibbs energy indicates a decrease in the adsorption rate at higher temperatures. This may due to the lower affinity of the adsorbent to the adsorbate at higher temperatures. A negative adsorption enthalpy indicates that the process can be described as exothermic. The release of additional energy (heat) may be due to the fact that the total energy released when the bond between the adsorbate and the adsorbent is formed is greater than the energy released when the bond breaks [65]. The adsorption enthalpy ($\Delta H^{0}$) of the sample adsorbent material for Pb^2+^ ions is smaller than that for Cu^2+^ ions, indicating that the material has a stronger affinity for Pb^2+^ ions [59]. The value of entropy ($\Delta S^{0}$) was found to be negative, indicating a decrease in randomness at the solid/solution interface. A negative value of entropy indicates that the adsorption process is driven by helium and exhibits reduced disorder during the adsorption process.

#### 3.6.6. Reuse Performance

**Figure 10:**
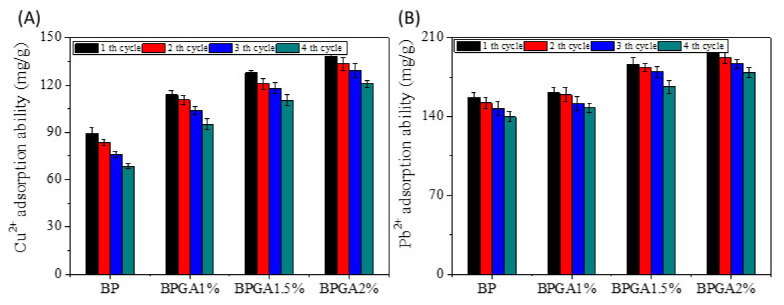
The reusability of the prepared adsorption material for Cu^2+^ and Pb^2+^ ions adsorption. (**A**) Cu^2+^, (**B**) Pb^2+^.

An ideal adsorbent not only has high adsorption capacity but also good reusability, which can improve efficiency and reduce cost. Therefore, desorption and reuse are very important for commercial application of composite adsorbents. Figure 10 shows that after the fourth cycle analysis, the regeneration rates of Cu^2+^ and Pb^2+^ by BP, BPGA1%, BPGA1.5% and BPGA2% were 76.69%, 83.67%, 86.42%, and 87.51% and 88.89%, 91.62%, 89.11%, and 90.85% respectively.

Thus, the prepared composite adsorbents exhibit good reusability and stability when Cu^2+^ and Pb^2+^ ions have been removed from aqueous solutions. Therefore, the prepared hydrogel BC/PVA/GO/APT can be used as a stable, environmentally friendly and highly efficient adsorbent.

## 4. Conclusions

The composite hydrogel BC/PVA/GO/APT is prepared by simple freeze-thaw cycles; a strong interaction hydrogen bond is formed between BC, PVA, GO, and APT. The introduction of GO and APT not only increased the specific surface area of the hydrogel, but also improves its thermal stability. The adsorption of Cu^2+^ and Pb^2+^ on the composite is spontaneous exothermic and is affected by the initial concentration, pH and temperature of the solution. The prepared hydrogel still has good Cu^2+^ and Pb^2+^ removal ability after four desorption and adsorption cycles. The composite was acid and alkali resistant and highly temperature resistant. Therefore, the prepared hydrogel BC/PVA/GO/APT can be used as an environmentally friendly and low-cost adsorbent for the removal of heavy metal ions or organic dyes from acidic or alkaline wastewater streams.

## Figures and Tables

**Table 1:** Adsorption kinetics parameters for the adsorption of Cu^2+^ ions onto the adsorbents.

Sample	Q_e_–Exp (mg/g)	Pseudo-First-Order			Pseudo-Second-Order			Intraparticle Diffusion Model	
		R^2^	Q_e,cal_ (mg/g)	k_1_ (min^−1^)	R^2^	Q_e,cal_ (mg/g)	k_2_ (mg/g min^−1^)	R^2^	K_t_ (mmol·g^−1^·min^−1/2^)
30 °C (Cu^2+^)									
BP	101.10	0.8750	86.89	0.0191	0.5309	102.56	0.82 × 10^−4^	0.9955	7.36
BPGA1%	109.55	0.9203	99.54	0.0179	0.6529	109.82	0.92 × 10^−4^	0.9984	7.17
BPGA1.5%	114.31	0.9448	105.27	0.0213	0.6154	113.33	1.1 × 10^−4^	0.9966	7.46
BPGA2%	120.79	0.9086	108.48	0.0215	0.6073	121.84	0.98 × 10^−4^	0.9986	7.70
40 °C (Cu^2+^)									
BP	59.39	0.9039	54.72	0.0186	0.6888	60.28	1.87 × 10^−4^	0.9964	3.48
BPGA1%	87.36	0.8802	77.53	0.0215	0.4864	91.00	1.24 × 10^−4^	0.9946	5.04
BPGA1.5%	97.01	0.8980	89.15	0.0231	0.5613	98.79	1.48 × 10^−4^	0.9974	5.27
BPGA2%	86.06	0.9981	86.06	0.0307	0.3634	88.27	3.65 × 10^−4^	0.9960	3.27
50 °C (Cu^2+^)									
BP	75.93	0.9514	72.69	0.0200	0.7543	76.13	1.98 × 10^−4^	0.9991	3.99
BPGA1%	105.22	0.9000	92.47	0.0296	0.3910	114.48	1.15 × 10^−4^	0.9931	5.71
BPGA1.5%	112.71	0.9369	101.02	0.0256	0.4685	117.75	1.11 × 10^−4^	0.9958	6.94
BPGA2%	114.15	0.9350	107.99	0.0245	0.5692	117.48	1.43 × 10^−4^	0.9959	6.00

**Table 2:** Adsorption kinetics parameters for the adsorption of Pb^2+^ ions onto the adsorbents.

Sample	Q_e_–Exp (mg/g)	Pseudo-First-Order			Pseudo-Second-Order			Intraparticle Diffusion Model	
		R^2^	Q_e,cal_ (mg/g)	k_1_ (min^−1^)	R^2^	Q_e,cal_ (mg/g)	k_2_ (mg/g min^−1^)	R^2^	K_t_ (mmol·g^−1^·min^−1/2^)
30 °C (Pb^2+^)									
BP	153.10	0.8372	122.55	0.0221	0.3726	153.42	0.44 × 10^−4^	0.9955	13.22
BPGA1%	160.39	0.8415	130.24	0.0249	0.3985	164.08	0.51 × 10^−4^	0.9957	12.76
BPGA1.5%	162.65	0.8765	137.07	0.0249	0.4275	166.18	0.58 × 10^−4^	0.9951	12.23
BPGA2%	167.69	0.9225	148.23	0.0253	0.4458	171.83	0.71 × 10^−4^	0.9931	11.56
40 °C (Pb^2+^)									
BP	154.21	0.9039	54.72	0.0186	0.8556	154.00	3.27 × 10^−4^	0.9993	3.81
BPGA1%	162.70	0.8802	77.53	0.0215	0.8125	164.54	3.31 × 10^−4^	0.9996	3.13
BPGA1.5%	163.19	0.8980	89.15	0.0231	0.7490	165.38	4.19 × 10^−4^	0.9998	2.54
BPGA2%	166.43	0.9981	86.06	0.0307	0.7963	167.27	4.97 × 10^−4^	0.9999	2.55
50 °C (Pb^2+^)									
BP	140.54	0.9878	140.35	0.0322	0.6870	140.05	2.92 × 10^−4^	0.9966	4.98
BPGA1%	145.99	0.9949	149.70	0.0321	0.7598	145.26	3.77 × 10^−4^	0.9985	4.05
BPGA1.5%	147.96	0.9880	151.16	0.0347	0.7675	147.67	4.18 × 10^−4^	0.9991	3.56
BPGA2%	154.58	0.9736	158.12	0.0351	0.7949	154.69	4.11 × 10^−4^	0.9992	3.36

**Table 3:** Maximum adsorption capacity of heavy metal ions on some materials.

Adsorbents	Heavy Mental Ions	Adsorption Capacity (mg/g)	Reference
BC	Cu and Pb	9.67 and 22.56	[60]
CMBC	Cu and Pb	12.63 and 60.42	[60]
CMC/GO	Cu	95.37	[61]
GO/CS	Pb	99	[62]
ATP/PES	Cu	25.3	[63]
SA/APT	Cu	119	[33]
This work	Cu and Pb	150.79 and 217.81	-

**Table 4:** Isotherm model parameters for the adsorption of metal ions onto the sample adsorption material.

Sample	Langmuir			Freundlich			Temkin		
	R^2^	Q_max_ (mg/g)	K_L_ (L/mg)	R^2^	K_F_ (mg/g)(L/mg)^1/n^	n	R^2^	K_T_ (L/g)	b_T_ (J/mol)
Cu^2+^									
BP	0.9040	-	-	0.9925	0.0116	0.54	0.9412	0.0256	32.7004
BPGA1%	0.9679	283.88	0. 0067	0.9987	4.9822	1.48	0.9745	0.1069	53.6006
BPGA1.5%	0.9319	315.49	0.0065	0.9972	5.3215	1.46	0.9563	0.1153	50.0054
BPGA2%	0.9981	-	-	0.9999	1.0525	0.99	0.9979	0.0417	30.4552
Pb^2+^									
BP	0.8891	-	-	0.9937	0.7393	0.78	0.9133	0.0675	24.7694
BPGA1%	0.9983	206.39	0.0937	0.9996	34.7633	2.44	0.9988	1.1320	60.7305
BPGA1.5%	0.6402	267.66	0.0326	0.9831	66.2883	5.12	0.8612	4.9339	81.6019
BPGA2%	0.6973	252.81	0.0926	0.9849	104.4456	12.08	0.8410	179.3227	124.7592

**Table 5:** Thermodynamic Parameters: change in Gibbs energy (ΔG^0^), in enthalpy (ΔH^0^), and in entropy (ΔS^0^) for Cu^2+^ and Pb^2+^ for temperatures between 303 K and 323 K.

Sample	ΔG^0^ (J/mol)			ΔH^0^ (J/mol)	ΔS^0^ (J/mol^−1^·k)		R^2^
	303 K	313 K	323 K				
Cu^2+^							
BP	−55.47	−44.11	−19.53	−21433.75	−72.18	0.7405	
BPGA1%	−482.63	−75.66	−280.88	−3892.62	−12.33	0.4360	
BPGA1.5%	−726.52	−155.59	−686.91	−1613.94	−3.81	0.5191	
BPGA2%	−1063.66	−730.51	−765.19	−6135.45	−18.42	0.2128	
Pb^2+^							
BP	−2981.85	−3161.13	−2310.79	−12982.00	−32.46	0.9928	
BPGA1%	−3525.01	−3834.60	−2671.68	−16214.46	−41.10	0.9898	
BPGA1.5%	−3707.99	−3877.31	−2807.23	−17154.83	−43.72	0.9931	
BPGA2%	−4150.19	−4167.74	−3291.18	−17021.82	−41.99	0.9971	
